# The use of serotonin reuptake inhibitors increases the risk of bleeding in patients with assist devices

**DOI:** 10.1186/s12872-022-02557-1

**Published:** 2022-03-22

**Authors:** Bianca Auschra, Markus J. Wilhelm, Claudia Husung, Josef Jenewein, Andreas J. Flammer, Lena Jellestad

**Affiliations:** 1 Department of Consultation-Liaison Psychiatry and Psychosomatic Medicine, University Hospital Zurich, Ramistrasse 100, 8091 Zurich, Switzerland; 2 Clinic for Cardiac Surgery, University Heart Center, University Hospital Zurich, Zurich, Switzerland; 3 Department of Cardiology, University Heart Center, University Hospital Zurich, Zurich, Switzerland; 4 Department of Medical Psychology and Psychotherapy, Medical University of Graz, Graz, Austria

**Keywords:** Serotonin reuptake inhibitors, Antidepressants, Bleeding, Ventricular assist device, Heart failure

## Abstract

**Background:**

Bleedings are frequent and dreaded complications in heart failure patients with ventricular assist devices (VAD). Serotonin reuptake inhibitor (SRI) antidepressants are widely used to treat depression in these patients, though they are attributed an increased risk of bleeding due to their modification of hemostasis. Evidence on bleeding risk of VAD patients under SRI medication is scarce and limited. We therefore aimed to assess if SRI use is associated with an elevated bleeding risk in this particularly vulnerable population.

**Methods:**

We analyzed the medical records of 92 VAD patients at the University Heart Center Zurich between September 2004 and April 2018 for the occurrence of bleedings and the concomitant use of an SRI. Bleeding was defined as any type of post-implantation bleeding requiring medical treatment. We performed univariate analyses and linear mixed-effects models, adjusting for baseline clinical characteristics as potential predictors to identify differences in bleeding rates in patients with vs. without SRI intake.

**Results:**

The cohort comprised 60.9% of patients with a continuous-flow VAD and 39.1% with a pulsatile-flow VAD. A total of 77.2% of patients experienced at least one bleeding incident. Overall, 28.6% of bleedings occurred under SRI therapy. A generalized linear mixed model showed a predictive effect of SRI medication on bleeding rate, independent of VAD type (*z* = 2.091, *p* = 0.037).

**Conclusions:**

Bleeding events in heart failure patients occur frequently after VAD implantation. Patients with SRI medication were at increased risk of bleeding. The indication and use of SRI, therefore, should be considered carefully.

## Background

Ventricular assist devices (VAD) may be used in patients with end-stage heart failure as a bridge to transplantation or destination therapy [1]. A major concern associated with the implantation of a VAD is thrombotic and hemorrhagic events. The contact of blood with the artificial inner surfaces and the turbulences generated by the pump lead to complex rheological changes and hemolysis through mechanical stress, which stimulates the activation and aggregation of platelets, increasing the risk for thrombus formation and bleeding [2]. Official guidelines recommend the administration of anticoagulant and antiplatelet drugs [3], however, these medications in turn increase the risk of bleeding, which makes balancing hemostasis in patients with VAD challenging [2]. The identification and management of concomitant risk factors, an optimized therapy of existing comorbid diseases and pharmacological adjustment is therefore of paramount importance in the clinical care of VAD patients.

Aside from its physical impact, heart failure, particularly in patients with a VAD, also takes a psychological toll. Approximately one in five patients with heart failure suffers from depression [4]. As a disturbed serotonergic neurotransmission plays an important pathophysiological role in depression [5, 6], serotonin reuptake inhibitors (SRI) as modulators of serotonin (5-HT) transmission belong to one of the key antidepressant drug classes. Owing to their favorable side effect profile, they are frequently used in depressed heart failure patients [7]. However, SRI have been associated with an increased risk of bleeding, likely caused by modification of hemostatic parameters and decrease of platelet activation and aggregation [8]. Thus, the combined administration of an SRI with anticoagulants [9, 10] or platelet aggregation inhibitors [11] might pose a particular risk of bleeding. VAD patients under anticoagulant and antiplatelet therapy with concomitant SRI medication therefore may have a particularly unfavorable risk profile for bleeding events. In cardiac surgery, to date, SRI use and risk of bleeding has mainly been investigated in coronary artery bypass grafting (CABG) patients, who did not show an elevated bleeding risk under SRI medication [12]. Evidence on bleeding risk and SRI intake in VAD patients is hitherto scarce and limited to reports on an increased risk of gastrointestinal bleeding (GIB) in left ventricular assist device (LVAD) patients under SRI [13] and serotonergic antidepressants [14], respectively.

In light of the discrepant findings of prior studies on bleeding risk under SRI treatment in cardiac surgical patients, this retrospective observational study aimed to shed light on the overall bleeding risk under SRI therapy in VAD patients and to compare bleeding rates in VAD patients with SRI therapy versus those without SRI therapy. We hypothesized that patients treated with SRI were more likely to suffer from bleeding events compared to patients without SRI treatment.

## Methods

### Study design and participants

We conducted a single-center retrospective cohort study on patients ≥ 18 years of age who underwent ventricular assist device implantation between September 2004 and April 2018 at the University Hospital Zurich. We consecutively included all patients with VAD treatment for at least two weeks who gave written informed consent, or, if already deceased, who did not have a documented disapproval to use health-related data for research. All patients had routine clinical follow-up at monthly intervals after discharge home. The follow-up period of data collection was not defined a priori—follow-up lasted until device removal (on recovery or transplantation) or death. Cases were administratively censored on Dec. 18, 2018. The study was approved by the national ethics committee (project ID 2018-00427) and was carried out in accordance with the Good Clinical Practice standards. The primary outcome was the occurrence of bleeding in VAD patients, defined as any type of post-implantation bleeding requiring medical intervention and/or treatment (e.g. blood transfusion, surgery, invasive interventions such as a gastroscopy, monitoring using diagnostic interventions such as computed tomography). Data were retrieved from a clinical database, including medical records on discharge notes and clinical follow-ups. In case of no bleeding event, information was assessed according to the last medical documentation. Following prior research [15], we excluded all bleedings that occurred within the first two weeks after implantation, assuming that bleedings in the first time period after implantation are primarily attributed to surgery related determinants obscuring the true effect of SRI medication on bleeding risk.

### Characteristics of ventricular assist device treatment

Clinical indication for VAD implantation was advanced heart failure according to INTERMACS level I – III [1]. Intention-to-treat was bridge-to-transplant in BiVAD patients, and bridge-to-transplant or destination therapy in LVAD patients. For left ventricular support, the Berlin Heart Incor LVAD was used from 2004 until 2008, afterwards the HeartWare LVAD became available. The Berlin Heart Excor BiVAD was used as biventricular assist device for both left and right ventricular support. For both LVAD and BiVAD implantation, a sternotomy was performed after which the patient was connected to the heart–lung machine. The inflow cannula was inserted into the apex of the left ventricle, and the outflow graft was connected to the ascending aorta. In BiVAD implantation, for an additional right ventricular support, an inflow cannula was implanted into the right atrium, and the outflow cannula was connected to the pulmonary artery.

Coumadin was used as oral anticoagulation (OAC) after LVAD implantation to achieve an INR between 2.3 and 2.8. In addition, aspirin 100 mg/day was applied for platelet inhibition. In the Berlin Heart Excor BiVAD, the INR was titrated at a higher level between 2.5 and 3.5 to prevent thrombosis in the right-sided circulation which has a lower blood pressure and, therefore, is more exposed to thrombosis. Like in LVAD patients, aspirin 100 mg/d was also administered in BiVAD patients. In case of bleeding, the OAC was stopped completely until the bleeding was under control and then medication was restarted.

### Clinical characteristics and medication

We reviewed all medical records for a documented bleeding and a concomitant intake of any SRI medication (see Table 1 for an overview of SRI classes and medication included) and other antidepressant agents.

**Table 1:** Overview of SRI classes and medications included in our sample

SSRI	SSNRI	Non-selective SRI
Sertraline	Venlaflaxine	Trazodone
Escitalopram	Duloxetine	
Citalopram		
Fluoxetine		
Paroxetine Vortioxetine		

SSRI = selective serotonin reuptake inhibitor. SSNRI = selective serotonin/norepinephrine reuptake inhibitor

Further, we reviewed records for demographics, etiology of heart failure and type of assist device, as well as concomitant medication (nonsteroidal anti-inflammatory drugs, OAC, antiplatelet therapy) and comorbidities (hypercholesterolemia, arterial hypertension, Diabetes mellitus type 2, chronic renal failure, nicotine abuse), which all may affect bleeding risk. We also collected data on a current diagnosis of depression and former depressive episodes, as well as the use of any antidepressant medication other than SRIs.

### Statistical analysis

Statistical analysis was performed using R 3.5.3. [16]. In univariate analyses, we used Chi-square tests for categorical and Mann–Whitney-*U* tests for continuous variables. Furthermore, we applied a linear mixed-effects model using the package *lme4* [17] to determine whether the occurrence of bleeding was predicted by administration of SRI medication or other antidepressant medication, or any other potential influencing factors (VAD model, arterial hypertension, hypercholesterolemia, chronic renal failure, nicotine abuse, Diabetes mellitus type 2). All reported *p* values were based on two-sided tests. A *p* value < 0.05 was considered statistically significant.

## Results

### Baseline characteristics

**Fig. 1:**
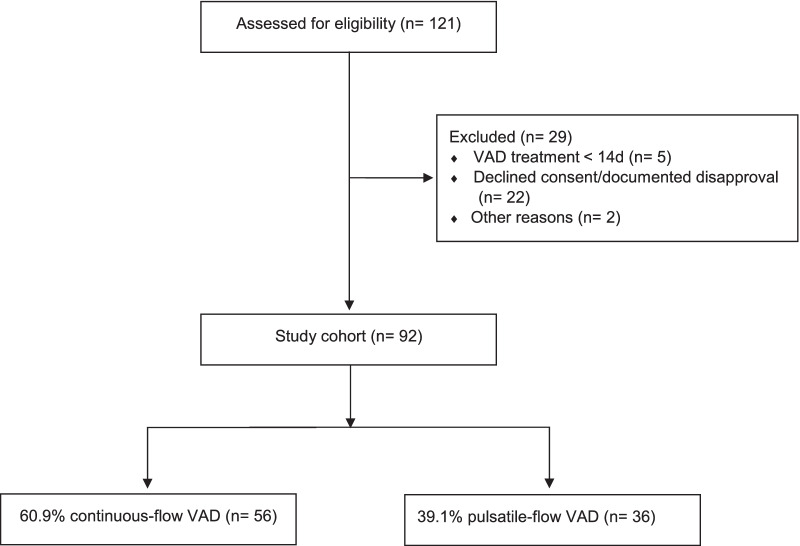
Consort diagram on VAD patients. Legend: VAD = ventricular assist device

Of the 121 patients who received a ventricular assist device, 92 patients met the inclusion criteria and were analyzed. The final cohort comprised 56 patients (60.9%) with a continuous-flow VAD and 36 (39.1%) with a pulsatile-flow VAD (Fig. 1). Two patients received serial implantation of two devices, in this case we considered only data from the first implantation for reasons of consistency. The median duration of VAD treatment was 185.5 days. The vast majority of patients (77.2%) suffered from at least one bleeding incident—17 patients (18.5%) experienced one bleeding and 54 patients (58.7%) experienced recurrent bleedings (> 1), with a median of three and a maximum number of 16 bleedings.

Univariate analysis demonstrated marginally significant associations between the occurrence of bleeding and both arterial hypertension (*p* = 0.093) and chronic renal failure (*p* = 0.086), all other demographic and medical variables showed no association. See Table 2 for baseline characteristics of the sample.

**Table 2:** Demographic and medical characteristics of the sample

	Global	Patients		*p* value
		With bleedings	Without bleeding	
	n = 92	n = 71	n = 21	
Age at time of implantation (median)	55.03	55.19	52.21	.199^a^
In years (range)	19.68–72.31	23.75–72.31	19.68–66.44	
**Gender**				.755^c^
Male	82.8%	83.1%	81.0%	
Female	17.4%	16.9%	19.0%	
**Hypercholesterolemia**	43.5%	40.8%	52.4%	.493^b^
**Arterial hypertension**	30.4%	25.4%	47.6%	.093^b^
**Chronic renal failure**	52.2%	57.7%	43.3%	.086^b^
**Diabetes mellitus type II**	25.0%	26.8%	19.0%	.575^c^
**Nicotine**				.277^b^
Current	23.9%	20.9%	33.3%	
Past	38.6%	37.3%	42.9%	
Never	37.5%	41.8%	23.8%	
**VAD Model**				.886^b^
Continuous-flow				
(HeartWare, Berlin Heart Incor)	60.9%	62.0%	57.1%	
Pulsatile-flow (Berlin Heart Excor)	39.1%	38.0%	42.9%	
**Type of cardiomyopathy**				.947^b^
Ischemic Cardiomyopathy	37.0%	38.0%	33.3%	
dilated Cardiomyopathy	35.9%	35.2%	38.1%	
Other etiologies	< 10% each	-	-	–
**Platelet aggregation inhibitors**				.689^c^
Dual	25.0%	22.5%	33.3%	
Mono	69.6%	71.8%	61.9%	
None	5.4%	5.7%	4.8%	
**Anticoagulants**	98.9%	100%	95.2%	–
NSAID (other than acetylsalicylic acid)	4.3%	5.6%	0.0%	–

Statistical analyses: ^a^ Mann–Whitney U-test, ^b^ Pearson’s Chi squared test, comparing patients with and without bleedings for each variable (in bold), ^c^ Fisher’s exact test. NSAID = non-steroidal anti-inflammatory drug. VAD = ventricular assist device

Four patients (4.3%) were diagnosed with a current depressive disorder, another three (3.3%) had at least one documented depressive episode in the past. 46 patients (50%) received antidepressant therapy, 26 patients (28.3%) received a SRI medication, 20 patients (21.7%) a non-SRI antidepressant medication. The group of non-SRI antidepressants comprised almost exclusively mirtazapine (95%). There was no standardized treatment protocol for antidepressant therapy. In part, the indication for antidepressant medication was based on recommendations of psychiatric specialists against the background of a diagnosis of depression or symptomatic treatment of corresponding clinical symptomatology (e.g., mirtazapine for insomnia), in part, it was initiated without specialist evaluation on the basis of presumed depressive clinical symptoms. In several patients antidepressant medication changed over time. Many patients experienced multiple bleedings, some under antidepressant medication and some without, thus all following analyses were based on bleeding events.

### Bleeding characteristics

A total of 401 bleedings occurred across the whole sample, on median 36 days (range 15–1295 days) after VAD implantation.

**Fig. 2:**
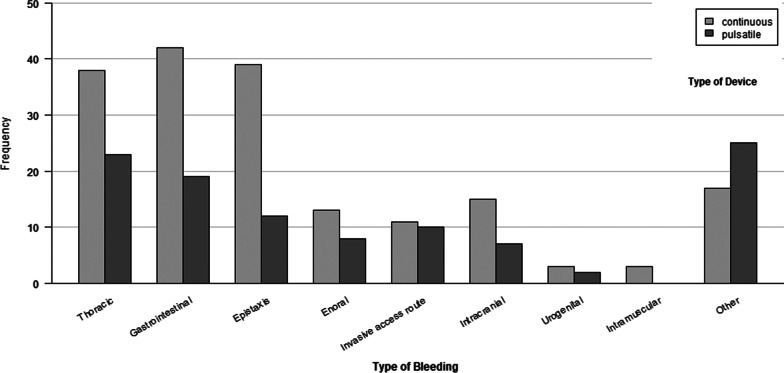
Absolute frequencies of bleeding by VAD type and bleeding category

Figure 2 provides an overview of absolute bleeding frequencies in the different bleeding categories by VAD type (pulsatile-flow and continuous-flow VADs). The majority of bleedings occurred in the three most frequent bleeding types (thoracic 21.3%, gastrointestinal 21.3%, epistaxis 17.8%), while all other types each contributed less than 10% of the total number of bleedings. Ratios of pulsatile-flow and continuous-flow VADs did not differ significantly between bleeding categories (*p* = 0.466).

In total, 42.5% of the bleedings were accompanied by an antidepressant medication. More specifically, 28.6% occurred under SRI therapy and 13.9% under antidepressants other than SRIs. As mirtazapine represented the almost exclusively used alternative antidepressant in our sample, we included it as a third group to compare patients on SRI medication with those receiving mirtazapine therapy and no antidepressant therapy at all.

A linear mixed-effects model with all the potential predictors, i.e. VAD model, arterial hypertension, hypercholesterolemia, chronic renal failure, Diabetes mellitus type 2 and nicotine abuse with patient as random factor showed a significant influence of SRI, but not of mirtazapine on the occurrence of bleedings, i.e., SRI medication elevated the risk of bleeding (*z* = 2.091, *p* = 0.037, while mirtazapine did not (*z* = 1.060, *p* = 0.289). Of the other potential predictors only the diagnosis of arterial hypertension showed a predictive effect on bleeding risk (*z* = 2.292, *p* = 0.022) (detailed results are listed in Table 3). Chronic renal failure remained a marginally significant predictor also in this comprehensive model. Anticoagulant and platelet aggregation inhibitor medication were not included in this model, as the ratio of patients not receiving them was too small to be analyzed (1% of patients). Non-steroidal anti-inflammatory drugs (NSAID) other than aspirin, in turn, were excluded as too few patients received them.

**Table 3:** Overview of fixed effects on bleeding rate

Groups	Estimate	Standard Error	Z value	*p*
Effect of SRI^1^	6.695	3.201	2.091	0.037
Effect of Mirtazapine^1^	9.96	9.391	1.06	0.289
**Covariates**				
Arterial hypertension	6.33	2.762	2.292	0.022
Chronic renal failure	− 3.849	2.214	− 1.739	0.082
Nicotine abuse	− 0.652	2.176	− 0.299	0.765
Diabetes mellitus type II	− 3.160	2.994	− 1.055	0.291
Hypercholesterolemia	2.184	2.175	1.004	0.315
VAD Model	0.604	1.204	0.501	0.616

^1^ compared to without antidepressant medication. SRI = serotonin reuptake inhibitors. VAD = ventricular assist device

A post hoc power analysis of the model revealed the power to be 0.832, indicating that the sample size was sufficient to reliably detect the effects reported above.

## Discussion

In this retrospective cohort study, we investigated bleeding rates in VAD patients with and without SRI medication. SRI use was significantly associated with the occurrence of a bleeding event, independent of VAD type.

As mirtazapine represented the almost exclusively administered alternative antidepressant in our sample, we included it as a third group to compare patients on SRI medication with those receiving mirtazapine therapy and no antidepressant therapy at all. In contrast to SRI medication, mirtazapine use did not lead to higher bleeding rates. In clinical practice, due to absent serotonin reuptake inhibition, mirtazapine has long been propagated as the safer alternative in patients with an elevated bleeding risk, which might also explain the frequent use in our cohort. While prior research also indicated a beneficial effect of mirtazapine regarding bleeding risks [18, 19], only recently a systematic review and meta-analysis of Na et al. pooled evidence on bleeding risk under mirtazapine and SSRI and suggested no priority of mirtazapine to SSRI [20].

Our investigation of the overall occurrence of bleedings in VAD patients expands upon prior evidence of bleeding risk under SRI medication in cardiac surgery, which until now predominantly encompasses coronary artery bypass grafting (CABG) patients. A recent meta-analysis of observational studies on CABG patients including seven studies and 146.811 patients did not find an increased risk of re-operation for bleeding under SRI medication [12]. Also, SRI intake did not increase the risk for any bleeding event after CABG surgery, even when accompanied by a concomitant antiplatelet [21] and antiplatelet/anticoagulant [22] therapy. In line with these findings, a large matched case–control study in cardiac surgical patients with cardiopulmonary bypass found no increased risk of bleeding under SRI medication [23]. Yet, given the distinct hemodynamics and complex rheological changes caused by a VAD, predisposing to pump thrombosis and thrombembolic events, VAD patients represent a particularly vulnerable patient group. OAC and antiplatelet medication are a mainstay of therapy in VAD patients to reduce this risk of thrombotic and cardiovascular events. However, their inherent risk of bleeding is a major concern, particularly in VAD patients, and special consideration of bleeding determinants in this population is warranted. Owing to their favorable cardiac risk profile SSRI are widely used in cardiac patients suffering from depressive disorders. Yet, they are potent inhibitors of platelet aggregation by reducing the uptake of serotonin into platelets [8]. As platelet activity essentially depends on the concentration of serotonin, a depletion of serotonin levels is associated with an increased risk of bleeding [8], which may critically affect VAD patients. In addition, some SSRI may increase the effect of OAC by inhibiting cytochrome P450 CYP (CYP) pathways, thus leading to a reduced elimination of OACs and a potentially higher bleeding risk [24]. Thus, the risk of bleeding in patients with a concurrent use of SRI medication and OAC/ antiplatelet therapy may be further elevated [9, 11]. In light of this, a careful consideration of concomitant pharmacological treatment is particularly essential in VAD patients.

Until now, however, there is only very limited research available on bleeding risk in VAD patients on antidepressant therapy, which comprises results on GIB only. Given our sample size, we were not able to analyze subgroups of bleedings, as analyses would have been underpowered. Thus, a direct comparison of results within the GIB category is not possible. However, our findings of an association with SRI intake and higher bleeding rates are in line with results of a recent multi-institutional retrospective study of Mawardi et al. of 248 LVAD patients, who reported an independent association of SSRI/SNRI therapy (including mirtazapine as mixed substance) with higher rates of GIB (OR 1.78, *p* = 0.045) [13] and a previous retrospective cohort study by Schultz et al., who also found a higher risk of GIB on serotonergic antidepressant therapy in their sample of 64 LVAD patients [14]. Different to our protocol, Mawardi et al. investigated solely post-discharge bleedings of patients with LVAD support of at least 100 continuous days [13]. Since in our sample a bleeding event occurred in median after 65 days, limiting the included patients to those with at least 100 continuous days of LVAD implantation, bleeding events in the early stage after implantation in patients with short-term VAD support would not have been considered. Also, in contrast to our design, they defined a minimum duration of SSRI/SNRI intake post-LVAD implantation of 30 days to qualify for the SSRI/SNRI group. As the start of SRI treatment holds a particularly high risk of bleeding [25], bleeding events in the early phase after SRI implementation might have been missed, even though they might even have accentuated the results. Moreover, bleedings in patients whose antidepressant treatment was discontinued within the first 30 days after VAD implementation (potentially due to an attribution of a bleeding event to an existing SSRI/SNRI medication) were not captured. Schultz et al., in turn, compared patients with all types of serotonergic antidepressant substances including tricyclic antidepressants and mirtazapine to a control group without antidepressant medication [14]. Due to the lack of data on the proportion of the different classes of serotonergic antidepressants in their sample, the ratio of SRI medication remains unclear. Also, we cannot derive information on whether—as in our study—the part of non-SRI antidepressants other than mirtazapine was similarly negligible. Since they, did not further differentiate between individual classes of antidepressants in a bleeding event, comparability to our results is limited. As most of the prior evidence in VAD patients, Mawardi et al. [13] and Schultz et al. [14] limited their research to LVAD patients. Our sample, however, also comprised BiVAD patients. While one might assume a potential impact on the results, our findings did not reveal a significant predictive effect of the type of VAD (pulsatile-flow vs. continuous-flow) on overall bleeding rate. Continuous-flow VADs have previously been associated with a higher risk in GIB compared to pulsatile-flow VADs [26]. A higher rate of AVM formation and acquired Von Willebrand syndrome is postulated as the underlying cause [27]. In our sample, however, we found no statistically significant difference in GIB ratios between VAD types, nor in any other bleeding category.

Aside from SRI medication, our analyses also indicated a predictive effect of a diagnosis of arterial hypertension. Management of arterial hypertension is of critical importance in clinical care of VAD patients and it has been linked to numerous adverse events like stroke [28] and pump thrombosis [29] in VAD patients. While our results generally support prior findings in VAD patients, which link arterial hypertension to adverse bleeding outcomes like hemorrhagic stroke [30] and intracranial hemorrhage [31], they contrast others, who found arterial hypertension not to predict GIB [32]. However, the retrospective design of our study with its inherent inaccuracies in documentation did not allow for an accurate ascertainment of blood pressure at the time of a bleeding event. Rather, only the diagnosis of arterial hypertension, as present at time of VAD implantation, could be recorded, which reduces the conclusiveness of our results in this respect.

Chronic renal failure showed marginally significant predictive properties with regard to an overall bleeding risk in our sample. This is consistent with previous evidence on a predictive potential of chronic kidney disease on bleedings (GIB) in VAD patients [32]. Concurrent cardiac and renal dysfunction, known as cardiorenal syndrome (CS), is frequent in chronic heart failure [33]. The rates of chronic renal failure in our sample correspond to existing evidence revealing that 45- 56.6% of heart failure patients show CS with at least moderate kidney dysfunction [34–36]. Also, the INTERMACS report of 2020 on 25,551 patients with LVAD support decribes a mean creatinin level of 1.4 mg/dl at time of VAD implantation [37], indicating frequent renal dysfunction and CS in chronic heart failure. SSRI intake, in turn, is associated with higher bleeding rates (GIB) in patients with renal dysfunction, further illustrating the complex and multi-faceted nature of increased bleeding risk in VAD patients on SRI medication [38].

The retrospective observational design of our study was subject to limitations regarding missing data and recording inaccuracies. The design did not allow to verify the dosage and duration of SRI medication, thus impeding a dose response analysis. However, at least in GIB, the dose of SSRI is not reported to have any effect on the risk of bleeding [25]. Owing to the size of subgroups, it was not possible to analyze the classes of SRI antidepressants (SSRI, SSNRI, SRI) individually. Moreover, potential underreporting of bleedings after discharge might have resulted in bleeding events being missed. Given the broad definition of our bleeding criterion and in light of the large variability of bleeding criteria in the existing studies, comparability of evidence on this topic is inherently limited. The retrospective design also did not allow a consistent rating of the severity of the individual bleeding on the basis of the available medical records. Indicative conclusions about the clinical relevance of bleeding can be drawn from the bleeding categories (e.g., epistaxis vs. intracerebral hemorrhage), however, subgroup analyses of bleeding categories were not possible as analyses were statistically underpowered, allowing only to present a general overview on bleeding frequencies within bleeding category by VAD types (Fig. 2). Reasons for this are limitations with regard to the statistical methodology used. We employed a linear mixed model to be able to take into account that the majority of patients experienced multiple bleedings. However, due to the structure of our data (e.g. a binary outcome variable) and the rather small sample size, our flexibility of representing our data in a comprehensive model was limited by the mathematical framework as implemented in the R package lme4, among others convergence criteria and imbalance of data [39]. This lead to promising results with high power, but prevented us from smaller post-hoc and subgroup analyses that could have broadened the insight provided. On the other hand, research shows that a model converging in lme4 yields outcome comparable to other mixed-model software once convergence is accomplished [39]. Designed as a monocentric study, our sample size in this specific group of VAD patients remains small, a limitation that should be overcome by future Studies on larger samples.

## Conclusions

In conclusion, the results of this study suggest higher bleeding rates in VAD patients under SRI medication, independent of VAD type. Mirtazapine, a widely used alternative antidepressant, in turn, was not associated with increased bleeding rates.

Our findings expand knowledge of a possible influence of SRI medication on bleeding risk in VAD patients, as our data encompass information on several bleeding types and both SRIs and mirtazapine. Even though our sample size was relatively small, our analyses might thus broaden the understanding of the influence of SRI on bleeding events and serve as a basis for further research. Our findings of higher bleeding rates under SRI therapy are consistent with previous research on individual (GIB) bleeding risks under SRI medication in VAD patients. While GI bleeding also accounted for a significant proportion of bleeding in our study, other specific bleeding entities in VAD patients on antidepressant therapy remain unstudied. While less frequent, intracranial hemorrhage in particular merits further investigation given its often critical impact on clinical outcome in VAD patients. Future studies with larger samples and a prospective design are needed to further assess the risk of concomitant antidepressant use in VAD therapy and to, ultimately, ensure an optimized treatment of this particularly vulnerable patient group. Undisputedly, VAD patients with depressive symptomatology should receive antidepressant medication when needed. However, physicians are advised to carefully weigh the use and choice of medication in awareness of bleeding risk. In selecting the appropriate medication, physicians should also consider alternative antidepressants without inhibition of serotonin reuptake.

## Data Availability

The data that support the findings of this study are available on reasonable request from the corresponding author. The data are not publicly available due to privacy or ethical restrictions.
